# Diagnostic Evaluation of Hypersensitivity Reactions to Antibiotics in a Large Cohort of Mastocytosis Patients

**DOI:** 10.3390/diagnostics13132241

**Published:** 2023-06-30

**Authors:** Jesper Jarkvist, Theo Gülen

**Affiliations:** 1 Department of Respiratory Medicine and Allergy, Karolinska University Hospital Huddinge, 141 86 Stockholm, Sweden; jesper.jarkvist@regionstockholm.se; 2 Department of Medicine Solna, Division of Immunology and Allergy, Karolinska Institutet, 171 77 Stockholm, Sweden; 3 Department of Medicine Huddinge, Karolinska Institutet, 141 52 Stockholm, Sweden; 4 Mastocytosis Center Karolinska, Karolinska University Hospital Huddinge, 141 86 Stockholm, Sweden

**Keywords:** mastocytosis, anaphylaxis, drug allergy, prevalence, antibiotics, beta-lactams, hypersensitivity, atopy, tryptase, KIT D816V

## Abstract

Background: Anaphylactic reactions are a well-known feature of mastocytosis, particularly in relation to hymenoptera venom stings. Although data on the frequency of drug hypersensitivity reactions is limited in mastocytosis, it is hypothesized that these patients may be predisposed to hypersensitivity reactions to certain drugs, including antibiotics. Nevertheless, this issue has not been systematically investigated. Thus, we investigate the prevalence and clinical features of hypersensitivity reactions to antibiotics (HRA) in mastocytosis. Methods: A 15-year retrospective study was conducted among 239 (≥18 years old) consecutive mastocytosis patients who were investigated in our center. All patients underwent a thorough allergy work-up, where self-reported reactions were individually evaluated by an allergist. Results: Overall, 34 patients (14.2%) were deemed to have HRA. Most patients reacted with cutaneous symptoms (74%), and anaphylaxis was rare, confirmed only in two of 34 patients (0.8%). Beta-lactams were the most common elicitors (63%). There were no differences in age, gender, atopic status and tryptase levels between mastocytosis patients with and without antibiotic hypersensitivity. Conclusions: The present study indicates a similar prevalence of HRA in mastocytosis compared to those of the general population. Moreover, antibiotics appear to be rare elicitors of anaphylaxis in these patients. Hence, our results suggest that mastocytosis patients without a history of HRA may be treated with these drugs without special precautions.

## 1. Introduction

Drug hypersensitivity reactions (DHRs) to antibiotics are a substantial problem for patients and physicians. As reported, antibiotics were implicated in 19.3% of all emergency department (ED) visits for drug-related adverse events in the United States [1]. Most DHRs to antibiotics consist of either immediate reactions, including pruritus, urticaria, vomiting, dyspnea, hypotension and anaphylaxis, or non-immediate exanthems of different severities [2]. Interestingly however, a true hypersensitivity to antibiotics can only be confirmed in about 10% of suspected cases [1,2]. With reference to risk factors, female patients as well as patients of increased age, most likely due to the increased number of cumulative exposures, appear to have an increased risk for DHRs to antibiotics [3].

Mastocytosis are a rare, heterogeneous group of disorders characterized by abnormal accumulation and activation of clonal mast cells (MCs) in different tissues, preferentially in the skin, bone marrow and gastrointestinal tract [4,5]. Mastocytosis can be systemic (SM) or cutaneous (CM) and in patients with monoclonal MC activation syndrome (MMAS), the World Health Organization (WHO) criteria for SM are not fully met [4,5]. In adult patients, mastocytosis tends to be persistent and presumably, inherent hyperreactivity and increased releasability of clonally aberrant MCs [6], which leads to provoked or unprovoked episodes of MC activation and anaphylaxis [7,8,9]. Elicitors of anaphylaxis in mastocytosis most commonly include the venom of hymenoptera stings [10], whereas food-induced anaphylactic reactions appear to be less common [11]. Moreover, unprovoked, i.e., idiopathic anaphylaxis episodes are also a common phenomenon in these patients [7,8,9,12]. Although data on the frequency of DHRs in mastocytosis patients is limited [13,14,15], it is generally believed that patients with mastocytosis may also be predisposed to a higher risk of severe DHRs to antibiotics, due to their inherently hyperreactive MCs. To support this notion, antibiotic-induced anaphylaxes have been sporadically reported in the literature. For instance, in a Spanish study analyzing elicitors of anaphylaxis in 163 adults with mastocytosis, antibiotics were the triggers of anaphylaxis in three cases (two cases with beta-lactams and one case after the administration of aminoglycosides) [16]. In another study, amoxicillin was identified as the cause of anaphylaxis in one patient [17]. Furthermore, in a case report of a patient with mastocytosis, amoxicillin was an elicitor of anaphylaxis [18]. Nevertheless, there are currently no studies systematically evaluating the prevalence and clinical features of DHRs to antibiotics in mastocytosis patients. Consequently, this issue has been an important source of anxiety among patients with mastocytosis, as well as physicians.

Overall, it remains uncertain whether there is a higher risk of developing DHRs to antibiotics with mastocytosis. Hence, the current study sought to investigate the prevalence and clinical characteristics of DHRs to antibiotics in a large cohort of mastocytosis patients. Furthermore, we analyzed whether the frequency and severity of these reactions are influenced by certain risk factors, such as serum baseline tryptase (sBT) levels, total IgE levels, atopic status, age, gender, or phenotype of MC disease. 

## 2. Methods

### 2.1. Study Population, Study Subjects and Study Design 

The Mastocytosis Center Karolinska was established in 2006 at Karolinska University Hospital in Stockholm, and 548 consecutive adult patients (≥18 years old) had been referred to the center due to suspected mastocytosis by 31 May 2021. The diagnostic workup included histopathological evaluations of bone marrow (BM) material, flow cytometry analysis, KIT D816V mutation analysis, and measurements of sBT levels. Of the patients investigated, a total of 268 obtained a diagnosis of mastocytosis according to current WHO-criteria [4,5,6]. Further, a 15-year retrospective study was conducted among 239 consecutive patients who provided their written consent to enroll in the study. Ethical approval was obtained from the Regional Ethical Review Board, Stockholm, Sweden (approval no. 2011/1750-31/3 and 2018/2621-31). 

Furthermore, all enrolled patients underwent a standardized allergy work-up at the Respiratory Medicine and Allergy outpatient clinic, including detailed medical history and allergy tests, e.g., a skin prick test (SPT) and/or specific IgE antibody tests (ImmunoCAP^®^), to assess the presence/absence of atopy [8]. Atopy was defined as a positive skin prick test result to at least 1 of the usually tested aeroallergens or a positive test result to the inhalant by Phadiatop^®^ (ImmunoCAP^®^, Thermo Fisher, Uppsala, Sweden), and atopic diseases were related to clinical symptoms among patients with atopic predisposition. Total IgE levels were measured in all patients, always together with sBT levels. Moreover, demographic data, history of anaphylaxis, the possible effects of general triggers, such as physical exertion, heat, cold, friction, emotional stress, alcohol, or histamine-containing food, were carefully evaluated. 

### 2.2. Diagnosis of Antibiotic Hypersensitivity 

Detailed clinical information regarding each patient’s history of reactions to antibiotics, time between drug intake and symptom occurrence (when available), clinical manifestations, and clinical outcomes were extracted from electronic patient charts. Patient-reported antibiotic reactions were identified, and these reactions were subsequently reevaluated in individual patients by an experienced allergist (TG) before classifying them as DHRs to antibiotics. Patients with isolated gastrointestinal symptoms or vague complaints were excluded. Furthermore, an allergy test (SPT and/or specific IgE) was performed based on patient history, i.e., in patients whose reactions occurred during the last 10 years. However, it is important to note that there are comparatively few validated antibiotic skin test procedures available, apart from beta-lactams. In addition, a more detailed review of the medical records was performed for individuals with possible antibiotic-induced anaphylaxis (AIA) to ensure that their anaphylaxis diagnosis was supported by the clinical findings and fulfilled the current criteria for anaphylaxis [19,20]. 

### 2.3. Drug Provocation Test

An oral provocation test to antibiotics was performed in only one patient. 

At that time, the patient was a 39-year-old male who was referred to our Center in February 2019 due to suspected mastocytosis after reacting during a phenoxymethylpenicillin (i.e., penicillin V) challenge at a local allergist. The patient had several penicillin (PCN) courses previously over the years due to recurrent sinus infections. On one such occasion during April 2017, he received a course of an oral penicillin V (Kåvepenin^®^, Solna, Sweden). On the eighth day of the treatment, he had redness in the face, felt a mild itchiness, and redness on his arms. However, he did not show symptoms of hives, swelling, or respiratory distress. The patient received H1 antihistamines with resolution of the symptoms and terminated the cure according to his GP’s advice. 

He was then referred to a local allergist and was investigated in September 2017 for a PCN V allergy. The skin prick test was negative for both PCN V and PCN G; however, specific IgE for PCN V was slightly positive (0.26 kU/L; ref. value < 0.10 kU/L), whereas PCN G was undetectable (<0.10 kU/L)). A PCN V challenge was carried out at the local hospital and about 10 min after receiving a tablet of Kåvepenin^®^ (800 mg), the patient started to feel unwell and lightheaded. Shortly thereafter he lost consciousness. His blood pressure was 60 mmHg systolic. He was immediately given intramuscular (i.m.) epinephrine 0.5 mg, and after 10 min received a further dose of 0.5 mg i.m. epinephrine followed by intravenous epinephrine to sustain blood pressure. He also received intravenous glucocorticoids (Solu-Cortef^®^, Sandwich Kent, UK) and hydration (Ringer-acetat Fresenius Kabi^®^, Halden, Norway). He was admitted to the emergency clinic for further observation and discharged after 24 h. Unfortunately, tryptase levels were first controlled at discharge, i.e., 24-h after the acute reaction, and they were slightly elevated (15 ng/mL; ref. value < 11.4 ng/mL). Since then, the patient avoided all beta lactams. A few months later, a follow-up visit at the local allergist revealed still-elevated sBT levels of 17 ng/mL. Thereafter, the patient was referred to our clinic for the investigation of a probable underlying mast cell disease due to elevated sBT levels and an unusual reaction pattern to the PCN V challenge despite a negative SPT and barely positive sIgE for PCN V. 

The patient underwent a comprehensive evaluation in our clinic. He had no history of atopy and did not report any symptoms of asthma or allergic rhinitis. The patient was never stung by bee or wasp during his adult years. Nonsteroidal anti-inflammatory drugs (NSAIDs), local anesthetics, and opiates were tolerated well. He had no history of anaphylaxis. Moreover, there were no unusual triggers as per his medical history on the day of the drug reaction; he ate his usual breakfast about 2 h before he conducted the PNC V challenge test. A SPT with commercial extracts (ALKNordic, Kungsbacka, Sweden) was performed, but did not reveal any sensitivity to standard aeroallergens, food allergens (cow’s milk, eggs, peanuts, hazelnuts, shrimp, fish, or wheat/rye/oat flour), hymenoptera venom (wasp or bee) and penicillin V and G. His total IgE level was 15 kE/L.

His physical examination was unremarkable; there were no signs of reddish/brown pigmented spots on the skin indicating urticaria pigmentosa. Furthermore, peripheral blood D816V mutation analysis was negative. Despite these findings, we decided to investigate with a BM biopsy due to his slightly increased sBT and severe, hypotensive reaction pattern during the PCN challenge. A histopathological evaluation of his BM biopsy revealed the presence of MC aggregates with atypical morphology, spindle-shaped MCs, and the presence of aberrant MCs expressing CD25 and CD2. The bone marrow aspirate was also positive for KIT D816V mutation. No other hematological disorder was found. Therefore, these findings fulfilled the diagnosis of indolent SM without skin engagement. There were no further anaphylaxis episodes during the four follow-up years. 

### 2.4. Statistics 

Statistical analyses were performed using SPSS 24.0 for Windows (SPSS Inc., Chicago, IL, USA), and a *p*-value of < 0.05 was considered statistically significant. Median values and ranges are presented to describe continuous variables and frequencies for categorical variables. Due to the fact that the distribution of these data was not normal, group differences were analyzed using the Mann–Whitney *U*-test. Categorical variables were analyzed with the Chi-Square test or Fisher’s exact test, when appropriate.

## 3. Results

### 3.1. Patient Characteristics 

Of 239 enrolled patients, 176 met the criteria for the diagnosis of SM according to WHO-criteria, whereas 18 patients had mastocytosis in the skin (MIS) and refused to undergo a BM-investigation. Additionally, 45 patients received diagnosis of MMAS, as they only fulfilled one or two criteria for an SM diagnosis. Demographics and characteristics of the enrolled subjects are demonstrated in Table 1. Patients with typical skin engagement of mastocytosis, i.e., urticaria pigmentosa, constituted 61% of SM patients (Table 1). Overall, the median age at diagnosis was 52 years, although this was lower in patients with MIS compared to other phenotypes (Table 1). Moreover, 93% of SM patients (164 of 176) had indolent disease.

Regarding bone-marrow findings, the presence of the major criterion, i.e., the MC aggregates, was found in 56.7% of SM patients. Furthermore, the median baseline tryptase for the overall cohort was 22 ng/mL (range 2.8–650) and levels were highest in the SM group. Atopy was present in 28.8% and the median total IgE levels were 16 kU/L with a wider range (1–1600). A history of anaphylaxis to known or unknown triggers was found in 51.5% of this cohort.

### 3.2. Prevalence of Hypersensitivity to Antibiotics

**Figure 1:**
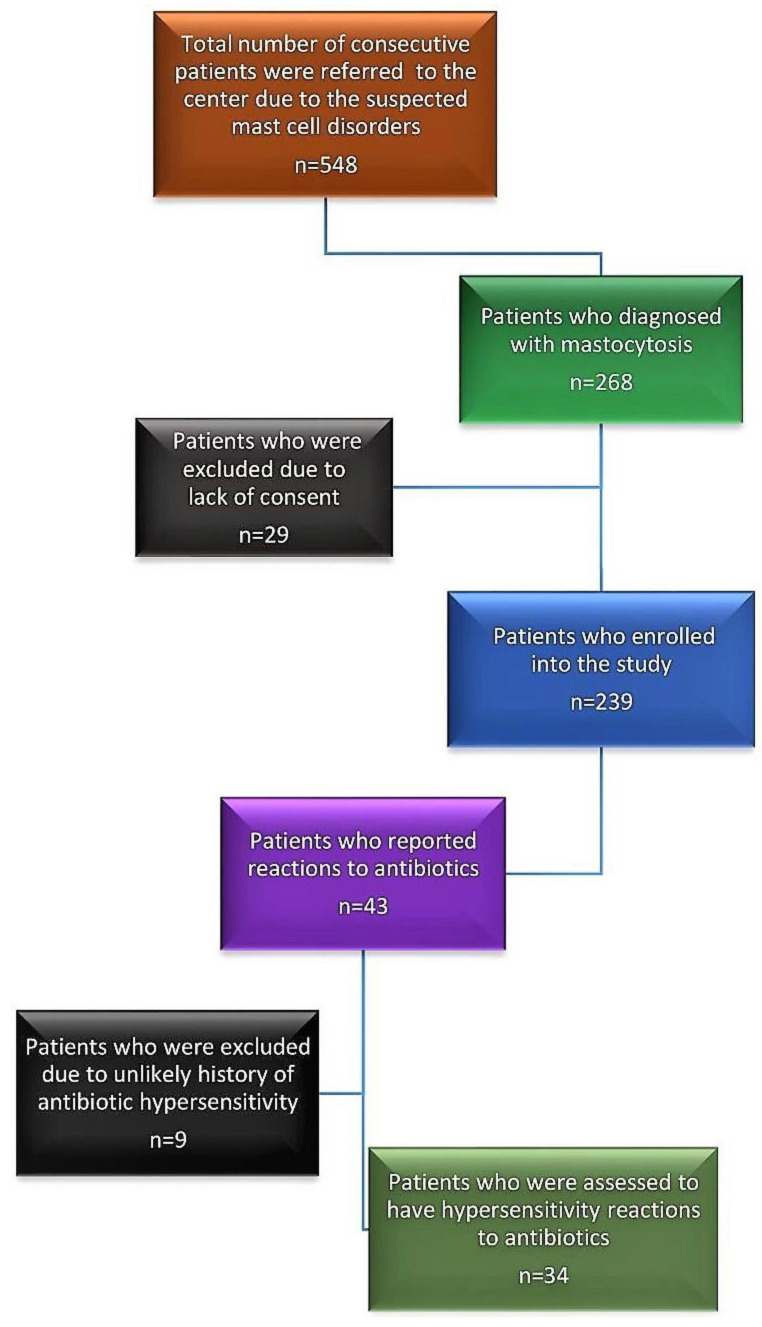
Flow-chart of the patient selection process.

Overall, 43 patients reported reactions to antibiotics; however, after individual clinical evaluations of patients by the allergist, only 34 patients (14.2%) were deemed to have a likely hypersensitivity reaction to antibiotics (Table 2). In addition, these patients had a total of 38 reactions, as four patients reacted against two different antibiotics. Frequency of antibiotic hypersensitivity was similar among patients with mastocytosis and MMAS (Table 2). Moreover, among patients with hypersensitivity, antibiotic-induced anaphylaxis was rare and confirmed in only two patients (Table 2). The flow-chart of this study is presented in Figure 1.

### 3.3. Clinical Features of the Reactions

**Figure 2:**
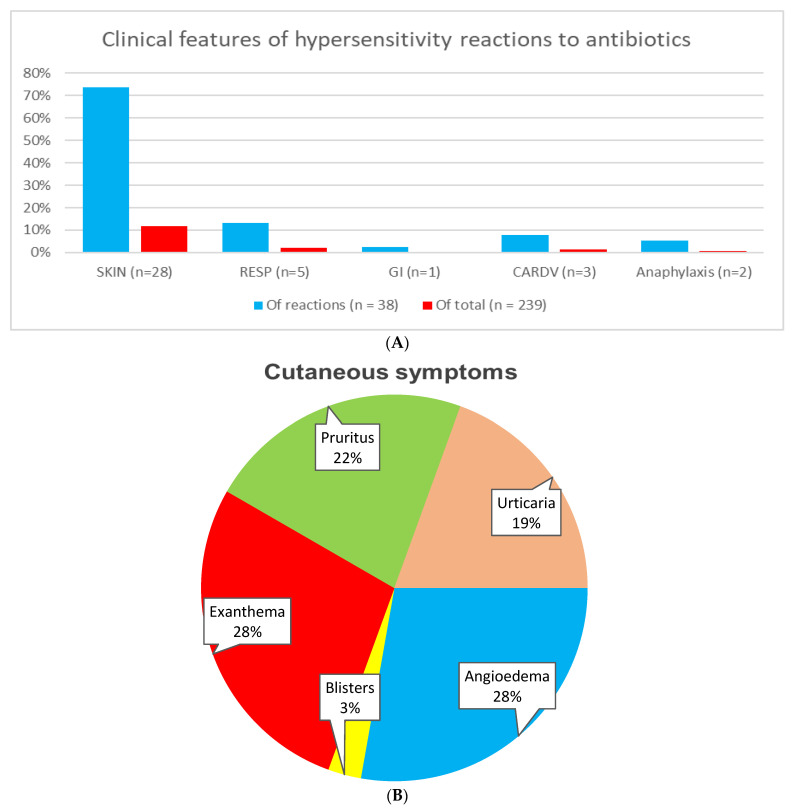
Clinical features of drug hypersensitivity reactions to antibiotics in mastocytosis patients: (**A**)—Involved organ systems during reactions. Of note, multiple organ systems may be involved in certain reactions. Abbreviations: GI, gastrointestinal; RESP, respiratory; CARDV, cardiovascular. (**B**)—Characteristics of cutaneous reactions. Notably, multiple skin-symptoms may be present during a single reaction.

The clinical patterns of antibiotic reactions are shown in Figure 2A. All antibiotic-related hypersensitivity reactions occurred before mastocytosis was diagnosed. Furthermore, no hypersensitivity reactions to antibiotics in patients with advanced SM were reported. The antibiotic reactions were mostly mild and limited to the skin, and immediate systemic hypersensitivity reactions were rare. Cutaneous symptoms were observed in 74% of all cases. Angioedema (28%) and exanthema (28%) were the most common skin symptoms, followed by pruritus (22%) and urticaria (19%) (Figure 2B). Respiratory symptoms occurred in 13%, cardiovascular symptoms in 8%, and gastrointestinal symptoms in 3% of reactions. Anaphylaxis accounted for 5% of overall antibiotic-induced hypersensitivity reactions (Figure 2A). Both patients with anaphylaxis had been diagnosed with ISM. Acute serum tryptase levels were not measured during anaphylactic reactions; however, both patients reacted with cardiovascular and respiratory symptoms within minutes of drug intake. 

### 3.4. Culprit Antibiotics 

**Figure 3:**
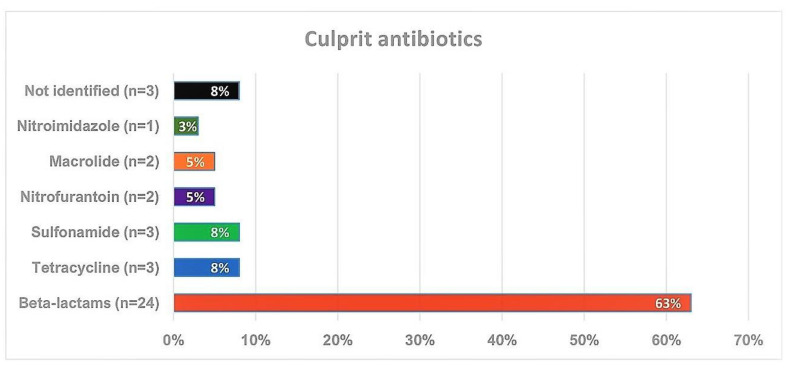
Distribution of culprit antibiotics eliciting drug hypersensitivity reactions (*n* = 38).

In this cohort, beta-lactams, in particular, penicillin, were the most frequent elicitors of the hypersensitivity reactions and accounted for 63%, followed by tetracycline (8%) and sulfonamides (8%) (Figure 3). Four patients reacted against two antibiotics on different occasions. In two cases, initial reactions were with sulfonamides and in a later occasion one patient reacted to penicillin V and another one to nitrofurantoin. In the third case, the patient initially reacted to penicillin V and in the second occasion to sulfonamides, and in the fourth case, the patient’s initial reaction was to macrolide, whereas the second reaction was to tetracycline. Furthermore, penicillin V (Kåvepenin^©^, Solna, Sweden) was the culprit drug in both anaphylactic reactions. In three cases, the culprit antibiotics were not identified, as the patients did not recall. Allergy tests (SPT and/or specific IgE) were performed in 18 of 24 (75%) patients with beta-lactam hypersensitivity. All patients tested negative except one who had an anaphylactic reaction to penicillin V during the drug challenge test. In the second anaphylaxis case, the reaction occurred several years prior and allergy tests were negative; therefore, the diagnosis was based on the patient’s strong medical history. 

### 3.5. Risk Factors of Antibiotic Hypersensitivity

Moreover, we investigated potential risk factors for developing hypersensitivity reactions to antibiotics. We demonstrated that total IgE levels were significantly higher among mastocytosis patients who reacted to antibiotics (*p* = 0.04). However, there were no differences regarding age, gender, bone-marrow findings, atopic status/diseases and sBT levels when we made comparison between mastocytosis patients with and without antibiotic hypersensitivity (Table 3). Of two patients with antibiotic-induced anaphylaxis, one was female and the other was male. Furthermore, in the MMAS patient group, all patients who reacted were females (*p* = 0.017). No other statistically significant risk factors were identified in MMAS. Interestingly, none of the patients who reacted with DHRs to antibiotics reported DHRs to other drugs. We further analyzed patients who had hypersensitivity to antibiotics and compared mastocytosis with MMAS patients. Baseline tryptase levels were significantly higher in mastocytosis patients compared to MMAS patients (*p* = 0.003). Nevertheless, age, gender, the presence of atopy, the presence of asthma/rhinitis, or total IgE levels did not significantly differ between groups.

## 4. Discussion

In the present study, we investigated the prevalence and clinical features of DHRs to antibiotics in a large cohort of patients with mastocytosis. An overall prevalence of antibiotic hypersensitivity was found to be 14.2%. Moreover, most reactions were mild and limited to skin; severe cutaneous and immediate systemic allergic reactions, i.e., anaphylaxis, were rare. Interestingly, all antibiotic hypersensitivity reactions occurred prior to a diagnosis of mastocytosis and no reactions among advanced SM patients were reported. To the best of our knowledge, this is the first study systematically investigating this complex issue in mastocytosis patients. 

Notably, the prevalence of antibiotic hypersensitivity estimated in this study is similar to the prevalence reported in large-scale studies in the general population, ranging from 10% to 20% [1,3,21,22]. A study of self-reported antibiotic allergy prevalence among 411,543 outpatients in San Diego County, California, found that 9.0% of patients had a penicillin allergy documented in their medical record [3]. Similarly, a retrospective study performed among 11,761 adult patients seen in the Internal Medicine Associates Clinic of Mount Sinai Hospital, USA, between 31 January 2012 and 31 July 2012, reported a history of penicillin allergy in 1348 patients (11.5%) [21]. In addition, another study described the prevalence of various drug allergies in the Greater Boston area, USA, documented in electronic health records among the total of 1,766,328 patients over two decades. More than one-third of the population had at least one drug allergy documented, including penicillin (12.8%) and sulfonamide antibiotics (7.4%) [22]. Notably however, the prevalence reported in this study is lower compared to some other patient groups. For instance, in patients with cystic fibrosis, an overall rate of hypersensitivity to antibiotics was reported to be 31% [23]. Thus, we may conclude that the extent of DHRs to antibiotics in mastocytosis patients does not differ significantly from the general population. 

With regard to clinical manifestations, DHRs to antibiotics were generally mild and limited to the skin in the present study. Thus, 74% of these patients presented with cutaneous symptoms, with angioedema and exanthema being the most common reaction patterns (28% each). The second most common symptoms originated from the respiratory system, which were found in 13% of the hypersensitivity cases. Furthermore, 5% of patients with antibiotic hypersensitivity were deemed to have anaphylactic reactions, and thereby the overall prevalence of antibiotic-induced anaphylaxis (AIA) was 0.8% in the study cohort. Moreover, when we compared the anaphylaxis rate to population studies, the results were of interest. Although large-scale studies investigating the actual prevalence of AIA in adults are limited, a study in the Boston area, USA, used the electronic health records of 1,756,481 patients and reported 19,836 cases of drug-induced anaphylaxis, corresponding to 1.1% of the population among the 622,152 patients with at least one reported drug hypersensitivity [24]. Moreover, in the same study, antibiotics have been reported to be the major group of drugs responsible for inducing anaphylactic reactions in 60% of cases (corresponding to an approximate 0.65% overall prevalence) [24]. Hence, the rate of AIA appears to be slightly lower in the general population when compared to our study. Nevertheless, another comparison can be made to the prevalence of nonsteroidal anti-inflammatory drugs (NSAIDs)-induced anaphylaxis in mastocytosis patients. Two recent studies with larger cohorts of mastocytosis patients reported a prevalence of NSAID-induced anaphylaxis to be 2.8% [25] and 9% [26], respectively, which are still three to eleven-fold higher than AIA. This finding is interesting since the overall prevalence of hypersensitivity to antibiotics and NSAIDs in mastocytosis is similar, reported to be 11% [25] and 13% [26], respectively; however, the clinical course of reactions with NSAIDs appear to be more severe than antibiotics in mastocytosis. Likewise, if we compare the risk of AIA to the risk of venom-induced anaphylaxis (VIA) in mastocytosis patients, there would still be a significant difference, as 28% of patients reacted to insect venoms in our study cohort. Therefore, the overall risk for AIA is 35-fold lower than for VIA in this cohort. Additionally, it is still threefold lower than food-induced anaphylaxis, as an overall prevalence of 2.5% was previously reported in mastocytosis patients [11]. 

Concerning the culprit agents in our study, beta-lactams were the most involved antibiotics, causing 63% of all hypersensitivity reactions and being responsible for both cases of anaphylaxis. This is in line with the studies evaluating antibiotic hypersensitivity in the general population in different settings, since beta-lactams, including penicillins, cephalosporins, carbapenems, and monobactams were the most common antibiotic classes reported to cause hypersensitivity reactions [21,22,27,28]. This may, to some extent, be reflected by patterns of antibiotic consumption, as beta-lactams are the most frequently prescribed drugs worldwide [29]. Beta-lactams are followed by sulfonamide antibiotics and tetracyclines in our study, each accounting for 8% of DHRs to antibiotics. Sulfonamides are also the second most commonly reported antibiotic hypersensitivity in the general population, which are documented in 2–10% of cases [21,22,27,28].

Considering risk factors with regard to drug hypersensitivity reactions in the general population, female gender and increasing age have been documented as risk factors in some previous reports [3,21,22,28,30,31,32]. Conversely, the role of atopy and atopic disease in DHR is controversial. However, a recent study, which evaluated the association of atopy with immediate-type beta-lactam allergies in 340 adult patients, found that determinants of atopy, total IgE, and specific IgE against mites were predictors of immediate-type beta-lactam allergies in a Spanish population [32]. In the current study, total IgE levels were significantly higher in mastocytosis patients who had DHRs to antibiotics compared to mastocytosis patients without a history of antibiotic hypersensitivity (*p* < 0.05). Nevertheless, we could not detect any risk factors related to gender, atopic predisposition, sBT levels, history of anaphylaxis or comorbidity with asthma and/or rhinitis, consistent with a previous report searching for an underlying risk for developing NSAID hypersensitivity in mastocytosis patients [25].

We acknowledge that the present study has certain limitations. Firstly, its retrospective nature is an inherent weakness. As the diagnosis of antibiotic hypersensitivity was mainly based on reported history and allergy tests followed by further assessments by an allergist, we cannot rule out is the possibility of a recall bias. The drug provocation tests to confirm or rule out diagnoses were not systematically performed, since most patients were reluctant to undergo such tests. We, therefore, excluded the cases with unreliable history. This might have altered the actual prevalence of hypersensitivity to antibiotics. An additional limitation is that despite the fact that most patients reported that the reactions occurred shortly after drug intake, in some cases, they did not recall the exact timing of the reaction. On the other hand, the main strength of our study is the ability to include data from a large cohort of well-characterized mastocytosis patients, as all enrolled patients underwent a standardized investigation protocol. In addition, this is first systematic study evaluating hypersensitivity to antibiotics in mastocytosis patients. Therefore, our data may provide important information regarding the frequency, severity and safety of antibiotics in these patients. 

In conclusion, the prevalence of DHRs to antibiotics appears to be similar in mastocytosis patients compared to the general population. Moreover, antibiotics were rare elicitors of anaphylaxis in these patients when compared to other triggers causing anaphylaxis, such as NSAIDs, venoms and foods. Based on our current results, we recommend that patients with a known tolerance to antibiotics continue using these medications without caution, whereas those with a prior reaction should undergo a thorough allergy work-up or use alternative drugs. Multicenter studies are, however, still required to ensure the reproducibility and generalizability of these results. 

## Figures and Tables

**Table 1:** Demographics and baseline characteristics of enrolled subjects.

Characteristics	Total (*n* = 239)	SM (*n* = 176)	MIS (*n* = 18)	MMAS (*n* = 45)
Male gender, *n* (%)	112 (46.9)	81 (46)	7 (38.9)	24 (53.3)
Age at diagnosis, median (range)	52 (18–84)	53 (18–84)	39 (18–76)	51 (28–74)
Presence of mast cell aggregates, *n* (%)	97 (46.4) 30 n/a	97 (56.7) 5 n/a	Not conducted	0 (0) 12 n/a
Presence of atypical morphology, *n* (%)	169 (81.3) 31 n/a	162 (95.3) 6 n/a	Not conducted	7 (21.2) 12 n/a
Presence of CD25, *n* (%)	200 (96.6) 32 n/a	169 (100) 7 n/a	Not conducted	29 (87.9) 12 n/a
Presence of D816V mutation, *n* (%)	164 (74.5) 1 9 n/a	135 (84.4) 16 n/a	9 (60) 3 n/a	20 (44.4)
Baseline tryptase (ng/mL), median (range)	22 (2.8–650)	30 (3.2–650)	11.5 (2.8–160)	10 (3.1–31)
Presence of atopy, *n* (%)	68 (28.8) 3 n/a	46 (26,3) 1 n/a	5 (29.4) 1 n/a	17 (38.6) 1 n/a
Presence of UP, *n* (%)	125 (52.3)	107 (60.8)	18 (100)	0 (0)
Total IgE (kU/L), median (range)	16 (1–1600)	14 (1–1 600)	13 (2.3–310)	47 (5.8–1 100)
Prescence of asthma/rhinitis, *n* (%)	59 (24.9) 2 n/a	40 (22.9) 1 n/a	3 (16.7)	16 (36.4) 1 n/a
History of any anaphylaxis, n (%)	123 (51.5)	89 (50.6)	0 (0)	34 (75.6)

Abbreviations: SM, systemic mastocytosis; MIS, mastocytosis in the skin; MMAS, monoclonal mast cell activation syndrome; UP, urticaria pigmentosa.

**Table 2:** Prevalence of antibiotic hypersensitivity in enrolled subjects.

Prevalence	Total Cohort (*n* = 239)	Mastocytosis (*n* = 194)	MMAS (*n* = 45)
Drug hypersensitivity to antibiotics, *n* (%)	34 (14.2)	29 (14.9)	5 (11.1)
Antibiotic-induced anaphylaxis, *n* (%)	2 (0.8)	2 (1)	None

Abbreviations: MMAS, monoclonal mast cell activation syndrome.

**Table 3:** Comparison of enrolled patients with or without antibiotic hypersensitivity.

Characteristics	Mastocytosis without Antibiotics Reaction (*n* = 167)	Mastocytosis with Antibiotics Reaction (*n* = 29)	*p*-Value	MMAS without Antibiotics Reaction (*n* = 40)	MMAS with Antibiotics Reaction (*n* = 5)	*p*-Value
Male gender, *n* (%)	78 (46.7)	10 (34.4)	0.12 *	24 (60)	0 (0)	0.017 *
Age at diagnosis, median (range)	53 (18–84)	46 (18–81)	0.259 †	51 (28–74)	54 (28–61)	0.820 †
Presence of atopy, *n* (%)	43 (25.8)	9 (31)	0.825 *	15 (37.5)	2 (40)	1.00 *
Presence of UP, *n* (%)	103 (62.2)	21 (72)	0.540 *	n/a	n/a	ND
Prescence of asthma/rhinitis, *n* (%)	35 (20.9)	9 (31)	0.349 *	13 (32.5)	3 (60)	0.336 *
Total IgE (kU/L), median (range)	13 (1–1600)	23 (1–250)	**0.048 †**	64 (5.8–1100)	22 (6.2–180)	0.234 †
Baseline tryptase (ng/mL), median (range)	29 (2.8–650)	28 (6.9–150)	0.474 †	10 (3.1–31)	10 (3.9–15)	0.636 †
History of any anaphylaxis, *n* (%)	73 (43.7)	16 (55)	0.557 *	32 (80)	2 (40)	0.085 *

Abbreviations: MMAS, monoclonal mast cell activation syndrome; MIS, mastocytosis in the skin; ND = not analyzed. * *p*-values were calculated using Fisher’s exact test; † *p*-values were calculated using a 2-tailed Mann-Whitney *U*-test; Bold indicates statistical significance (*p* < 0.05).

## Data Availability

The data are not publicly available due to privacy or ethical restrictions. The data that support the findings of this study are available on request from the corresponding author.
